# Family studies to find rare high risk variants in migraine

**DOI:** 10.1186/s10194-017-0729-y

**Published:** 2017-03-02

**Authors:** Rikke Dyhr Hansen, Anne Francke Christensen, Jes Olesen

**Affiliations:** Danish Headache Center, Department of Neurology, Rigshospitalet Glostrup, University of Copenhagen, Glostrup, DK-2600 Denmark

**Keywords:** Next generation sequencing, Common complex disease, Whole genome sequencing, family approach, Whole exome sequencing, Migraine genetics

## Abstract

**Introduction:**

Migraine has long been known as a common complex disease caused by genetic and environmental factors. The pathophysiology and the specific genetic susceptibility are poorly understood. Common variants only explain a small part of the heritability of migraine. It is thought that rare genetic variants with bigger effect size may be involved in the disease. Since migraine has a tendency to cluster in families, a family approach might be the way to find these variants. This is also indicated by identification of migraine-associated loci in classical linkage-analyses in migraine families. A single migraine study using a candidate-gene approach was performed in 2010 identifying a rare mutation in the TRESK potassium channel segregating in a large family with migraine with aura, but this finding has later become questioned. The technologies of next-generation sequencing (NGS) now provides an affordable tool to investigate the genetic variation in the entire exome or genome. The family-based study design using NGS is described in this paper. We also review family studies using NGS that have been successful in finding rare variants in other common complex diseases in order to argue the promising application of a family approach to migraine.

**Method:**

PubMed was searched to find studies that looked for rare genetic variants in common complex diseases through a family-based design using NGS, excluding studies looking for de-novo mutations, or using a candidate-gene approach and studies on cancer. All issues from Nature Genetics and PLOS genetics 2014, 2015 and 2016 (UTAI June) were screened for relevant papers. Reference lists from included and other relevant papers were also searched. For the description of the family-based study design using NGS an in-house protocol was used.

**Results:**

Thirty-two successful studies, which covered 16 different common complex diseases, were included in this paper. We also found a single migraine study. Twenty-three studies found one or a few family specific variants (less than five), while other studies found several possible variants. Not all of them were genome wide significant. Four studies performed follow-up analyses in unrelated cases and controls and calculated odds ratios that supported an association between detected variants and risk of disease. Studies of 11 diseases identified rare variants that segregated fully or to a large degree with the disease in the pedigrees.

**Conclusion:**

It is possible to find rare high risk variants for common complex diseases through a family-based approach. One study using a family approach and NGS to find rare variants in migraine has already been published but with strong limitations. More studies are under way.

**Electronic supplementary material:**

The online version of this article (doi:10.1186/s10194-017-0729-y) contains supplementary material, which is available to authorized users.

## Background

With a lifetime prevalence of 16%, migraine affects 75 million Europeans. It can be very disabling for the individual and is a large economic burden to society [1]. Unraveling the genetics of migraine is therefore highly relevant. Migraine is a complex disorder caused by several genes and environmental factors [2, 3]. A higher concordance of migraine in monozygotic than in dizygotic twins, and the 1.9-3.8 fold higher risk of migraine among first degree relatives of affected individuals, indicates an important genetic component [2–5] Twin studies show a heritability of 34%–65% [5, 6]. Heritability of migraine with typical aura (MA) that affects approximately one third of migraineurs is higher than for migraine without aura (MO), and evidence supports that MA and MO have different, though somewhat overlapping, etiology [7, 8]. The diagnosis of migraine is based solely on patients’ history in the absence of validated biomarkers.

Causal mutations in three genes have been identified for familial hemiplegic migraine (FHM), a rare and severe, autosomal dominantly inherited subtype of migraine with aura [9–11]. However, these genes are apparently not involved in the prevalent types of migraine [12, 13]. Several linkage studies and association studies using a candidate-gene approach have failed to identify any robust association between genetic variants and the prevalent types of migraine (see Table 1 for explanation of terms and methods mentioned) [14]. Recently, a large meta-analysis including almost 60,000 affected subjects demonstrated 44 independent common single nucleotide polymorphisms (SNPs) associated with migraine [15]. Odds ratios (ORs) ranged between 0.85 and 1.11, and the mechanisms of action in migraine are unknown. It is now clear that no common variants are associated with a medium or high risk of migraine. Thus, common variants cannot explain much of the observed heritability of migraine. In general, only 1.5%–50% of heritability of common complex diseases and traits can be explained by common variants [16]. In other words, variants with medium or high risk must be rare and therefore not possible to capture by genome-wide association studies (GWAS) in unrelated case–control samples [17]. Migraine sometimes clusters in families with an inheritance pattern that often looks dominant. MA in particular seems to aggregate in large families [2, 3]. It is reasonable to think that rare genetic variants or mutations with medium to high effect size play an important role in these large families [18]. The same could be the case for families with multiple individuals affected by MO. A search for rare variants conferring a medium to high risk of migraine should therefore focus on families with many affected with migraine. Thus, it is hypothesized that the prevalent types of migraine can be oligogenic or even monogenic inherited, as is also hypothesized for many other common complex diseases [18]. If oligogenetic inherited, susceptibility is due to a specific combination of rare variants and unrelated cases represent a wide range of different combinations. These would be impossible to capture by GWAS alone. In a family, the combination of variants is probably specific to that one family, and because of this clustering of variants it will increase the chance to find it. Linkage analysis is the method of choice when studying monogenic disorders, but linkage studies are difficult in case of oligogenic inheritance. Previous linkage analyses in migraine families have shown association between several loci and MA and MO with LOD > 3 [19–25]. None of these associations have been consistently replicated and the causal genetic variants remain to be identified, but it shows that the family approach is promising for migraine. Based on an a-priori hypothesis about the involvement of the TRESK potassium channel (encoded by the KCNK18 gene) in MA, Lafreniére et al. 2010 sequenced the entire gene region (i.e. a candidate-gene approach) and identified five variants in a case–control sample, of which one rare variant was subsequently shown to segregate perfectly with MA in a large multigenerational family (eight affected and 8 unaffected members) [26]. However, this association has later become questioned [26–28]. The technology of next-generation sequencing (NGS) now provides an affordable tool to investigate genetic variation in the entire exome or genome [29]. In theory, a family based study design and genome sequencing can embrace the search for rare variants with both high and medium effect size, whether monogenic or oligogenic. The family approach using NGS has not yet been used in migraine genetic research on a larger scale, but we and probably several other groups now have ongoing studies. We therefore judge it timely to investigate how the family approach and NGS has been used successfully in other common complex disorders, and to deduce from that the possibilities for its application in migraine.

**Table 1:** Explanation of genetic methods and terms

Method/term	Description
Single nucleotide polymorphism (SNP)	A SNP is a substitution of a single base pair in the genome that occur in >1% of a population, a so called common variant [84, 85]. SNPs that occur in <1% of a population are considered rare. On average, there is one SNP for every 0.75-1.91 kb throughout the genome [86, 87]. Many of these reside outside protein-coding areas. A proportion of these will reside in other functional elements [88]. <1% of SNPs lead to changes in protein function [89]. After completion of especially the HapMap project and The 1000 Genomes Project, the vast majority of SNPs and structural variants are now mapped throughout the genome [86, 87, 90–92]. More than 38 million SNPs are identified and these are estimated to constitute more than 95% of all common SNPs [91]. The SNPs known to date are gathered in public databases like dbSNP [33] .
LOD-score	LOD = logarithm of the odds. A measure of the probability of two genetic loci to be located close to each other on a chromosome and thereby the likelihood for them to be inherited together (be linked). A LOD-score on > 3 means that the likelihood for two loci to be located close (and be linked) is 1,000 times the likelihood of no linkage [93].
Genome wide association study (GWAS)	The rationale is to find variants that happen to occur more often than by chance in the genomes of individuals with a specific phenotype. It is carried out by an association analysis on genotyped cases and controls. SNPs are most widely used as genetic marker. Genomes are genotyped at specific points in the DNA where the chosen markers are localized if present. Every SNP represents a block of genes, a haplotype. These are inherited together more often than by chance. They are said to be in linkage disequilibrium [85]. Tag-SNPs present in the sample are tested for association with a phenotype of interest, e.g. migraine, by comparing the frequencies of the SNPs in cases vs. controls.
Nest generation sequencing (NGS)	Sequencing of the nucleotides in the entire exome or genome by whole exome or whole genome sequencing (WES or WGS, see below)
Whole exome sequencing (WES)	WES is sequencing of every nucleotide in all exomes in a genome. Exomes are the protein coding part of DNA. This means that the remaining part of DNA in between the exomes is not sequenced.
Whole genome sequencing (WGS)	WGS is complete sequencing of the entire genome consisting almost 3 billion base pairs [89]. Thus, also non-coding parts of the DNA are sequenced. Non protein coding DNA contains many functional elements with influence on gene expression and regulation e.g. RNA coding sequences, transcription factor binding sides, regions of modification or with influence on chromatin (the DNA, RNA and proteins that chromosomes are made of) structure and other interacting regions [88].
Linkage-analysis	Attempts to find chromosome segments that are shared between affected family members. Thus, no prior hypothesis of involved loci is needed. To screen for shared DNA blocks, markers are needed. Often, sets of microsatellite-markers are used [94]. Microsatellites contain a short sequence of base pairs that are repeated a variable number of times. Every microsatellite represents a block of DNA, a haplotype. Thus, having a specific microsatellite means having a specific haplotype. The aim is then to find linkage between a phenotype e.g. a disease and a haplotype. If a haplotype segregates with a disease in a family, they are probably linked.
Haplotype	Each gene has a specific position on a chromosome, a so called locus. A haplotype is a combination of gene alleles at a chromosome that are inherited together more often than by chance. On average haplotypes span 25,000 nucleotides [84, 85]. Haplotypes are longer for newer and inbred or isolated populations and shorter for old or very outbred populations [91].
Sanger sequencing	A classic method to sequence every nucleotide in a DNA fragment of interest. The method includes the use of modified nucleotides labeled radioactively or by fluorescence and gel electrophoresis [95]. More precise sequencing with fewer read errors that WES/WGS. It is used to confirm findings in WES/WGS.
Phasing and imputation	Imputation is performed with different kinds of software and is a way to predict not genotyped variants, located between genotyped variants in haplotyped blocks, by using a reference sample where a greater number of variants are genotyped [96]. Phasing means to sort out which genotypes are placed on the paternal respectively the maternal chromosome [97].
Identity by descent (IBD)	Genomic regions that are identically inherited from parents to more than one child. This means that the siblings will share the DNA combination in that region [63]. IBD can prevail over many generations and reveal the familial relationship (a common ancestor) between very distantly related individuals.

## Method

This is a focused review and by no means exhaustive on the topic of NGS in common complex diseases.

PubMed was searched to find studies on rare genetic variants using NGS in common complex diseases with positive findings.. There is no clear definition of the term “common disease”. The definition of a “rare disease” is not clear either, but it is defined by the European Commission as “*prevalence of less than five per 10,000 in the Community*” [30]. We therefore defined diseases not fitting this definition as common. Successful studies with a family-based design using a NGS method, not looking for de novo mutations or already known candidate genes were included. Studies focusing on cancer were also excluded. We searched the following terms: “Exome AND sequencing AND pedigree AND rare AND variants” which resulted in 192 articles of which 29 abstracts were read, 15 were read in full and 14 were included. Also the terms “family-based AND “exome-sequencing” AND rare AND variants” were searched and yielded 17 articles (three already included) out of which nine abstracts were read and eight articles were read in full resulting in three included studies.

“Migraine AND “whole genome sequencing ”and “migraine AND” “whole exome sequencing” only yielded three and nine hits respectively and one article were read in full and included.

We especially wanted to include studies of bipolar disorder and schizophrenia, because the diagnosis, like for migraine, relies on clinical characteristics in lack of a biomarker. Therefore, we also searched the terms “Schizophrenia AND “whole genome sequencing” AND families” yielding six articles whereof two were included, “Schizophrenia AND “whole exome sequencing” AND families” yielding 13 articles (one already included) of which 8 abstracts were read, 5 of them in full resulting in 3 included. The terms ““bipolar disorder” AND “whole exome sequencing” AND families” resulted in one article which was not included and ““bipolar disorder” AND “whole genome sequencing” AND families” resulted in two articles of which one was included.

The last search was performed the 12^th^ of June 2016.

**Fig. 1:**
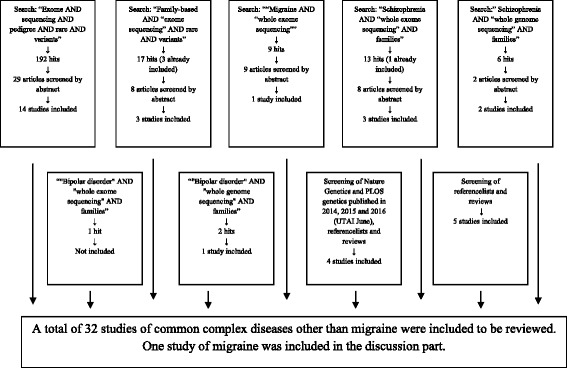
Flowchart of the searching process

We expected highly relevant articles on the topic would be published in Nature Genetics and PLOS genetics. Therefore every issue of the two journals published in 2014, 2015 and 2016 (UTAI June), were screened for relevant papers. This and reading through reference lists of reviews and other relevant papers lead to inclusion of another 10 studies. A total of 32 studies were included (Fig. 1).

## Methodology of the family approach

In the following we shall describe a promising design for a family-study to find rare high risk variants in complex diseases used in our ongoing studies. For explanation of the different methods and terms mentioned, see table 1.

### Inclusion

Multigenerational pedigrees with as many affected and unaffected individuals as possible will optimize the chance to find causal variants. The affected family members have to be in direct bloodline, with a minimum of affected spouses. There must be a trio of an affected child and an affected as well as a non-affected parent and also an affected relative in direct blood line as many meioses away as possible to narrow down the number of shared variants.

### Diagnosis

To distinguish affected individuals from unaffected, the diagnostic process is of crucial importance. A single wrongly diagnosed individual in a family, whether affected or unaffected, will diminish the chance to identify the causal genetic variant(s) in the segregation analysis. For migraine this is difficult as no diagnostic biomarker exists. The diagnosis relies on a detailed recording of symptoms and unambiguous diagnostic criteria of the International Headache Society (international classification of headache disorders third edition (ICHD 3-beta) [31]). A validated semi-structured interview based on the diagnostic criteria, allows a reliable diagnosis. The interview should be performed by a specially trained physician or senior medical student. An example is the validated, semi-structured migraine interview used at the Danish Headache Center [32].

### Molecular genetics

Whole exome sequencing (WES) or preferably whole genome sequencing (WGS) can be performed on DNA from blood samples or cells from other biological materials like mucosal cells from a buccal swab. The analysis will be a combination of an association- and a segregation analysis which will require a combination of several sequencing and genotyping methods. Based on an in-house protocol and literature review we propose the following.

**Fig. 2:**
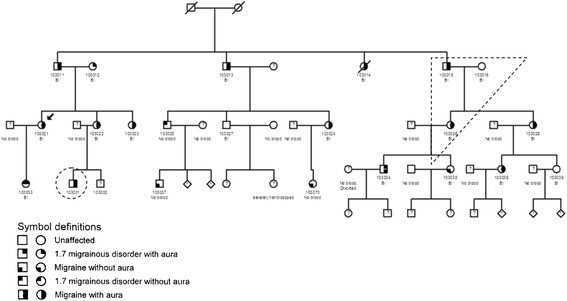
Example of a MA family suited for a family approach analysis. A trio and a distantly related, affected family member are marked with *dashed lines*. The *arrow* marks the proband

A so called family trio is selected from each pedigree for sequencing (in some cases it can be preferable to only sequence the proband). WGS is optimal because causal genetic variants can be localized in non-protein coding regions. WES may be chosen because of the lower cost. A trio composed of a nuclear family with an affected child, an affected parent, a non-affected parent (Fig. 2) and a distantly related affected family member from the same pedigree as many meioses from the trio as possible are sequenced.

Several hundred variants are usually shared between the affected individuals. The disease-causing variants will often not be present in DNA from the unaffected relative. The use of a more distantly related affected individual in the analysis will decrease the number of possible causal variants because a distantly related person shares less DNA with the two affected individuals in the trio. Quality testing of the sequencing like depth of coverage (the a verage number of times a nucleotide is expected and observed to be sequenced) will not be explained here in details, but the importance of this step should be noted.

### Filtering

To narrow down the number of variants, a filtering process is hereafter necessary. This can be done in several ways. They all depend on several existing databases with genetic information. These databases consist of collections of structural variants like SNPs and copy number variants (CNVs) detected by different studies. All SNPs known to date are for example gathered in public databases like dbSNP [33]. Other examples are the 1000 genomes project data, the Database of Genomic Variants [34], and more local databases like LuCamp containing 700.000 SNPS from 2000 Danish individuals [35]. The strategy is to filter out and exclude all variants known in all available genetic databases as the causal variants are expected to be private for a family. Some studies choose to include rare variants present in databases with minor allele frequency (MAF) <0.5% or <0.1% [36]. A third strategy is to filter out variants with e.g. MAF >0,1%, thereby excluding variants common in the population from the study data [37, 38].

### Co-segregation studies

The remaining family specific variants then have to be tested for segregation with disease in the pedigree. This can be done by a classical Sanger sequencing of the relevant genes in all the non-sequenced family members or by SNP genotyping. Ideally the causal variant is present only in affected individuals, but the possibility of unaffected carriers has to be taken into account because of incomplete penetrance, or later debut of the disease. Calculating a so called LOD score (LOD = logarithm of the odds), can help as a measure of probability of linkage between a variant and a disease.

Oligogenic inheritance where more than one variant are found to segregate in a pedigree is very likely in common diseases. Therefore, it is possible that this approach will result in multiple variants segregating in one family, all playing a role for the pathogenicity in that family.

### Further analyses

**Fig. 3:**
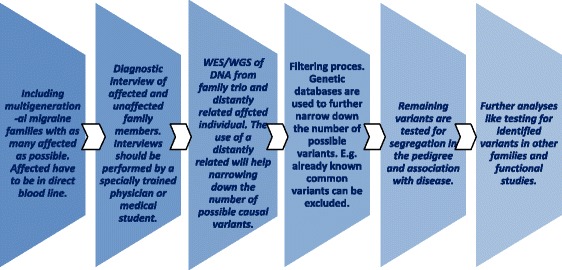
Description of the family approach in summary

Further analysis, like testing whether some of the segregating variants are shared between different families within the same study is interesting. The likelihood of a variant to be disease-causing can be tested by screening a sample of unrelated cases or additional sets of affected families for the mutation [36]. Screening for other mutations in the involved genes is also relevant. A so called burden test can be done. The load of rare mutations in a gene or gene family present in cases vs. controls reflects the probability of a gene to be involved in disease pathogenesis. Afterwards, functional studies involving laboratory tests are crucial to investigate how detected variants are involved in pathogenic mechanisms of the disease of interest. Different kinds of software like polyphen-2 [39] and SIFT [40] have been developed to predict to which extent a variant or mutation is functional. This prediction is based on the possibility of a variant to be e.g. deleterious or in-frame. Laboratory tests may show whether the mutation resides in a gene (this will always be the case when using WES) or in a regulatory area. Other relevant questions to ask are: in which processes or pathways is the variant involved? Does this affect gene or protein expression or function? Can this explain development of symptoms of the investigated disease? Functional laboratory studies in cells from humans or animals can help answer these questions (Fig. 3).

## Successful use of the family approach in diseases other than migraine

Family studies using different variants of the approach described above or some of its elements have already provided interesting results in common complex diseases other than migraine.

Thirty-two studies that fulfilled the criteria were included in this review [36–38, 41–69]. Six of them studied bipolar disorder, five of them studied schizophrenia (SCZ), three of them autism spectrum disorder (ASD) and two of them type II diabetes, while four studies focused on late onset Alzheimer’s disease (LOAD). There was one study focusing on each of the following: Parkinson’s disease, age-related macular degeneration (AMD), adiponectin level, atrial fibrillation, intracranial aneurysms, nonsyndromic cleft lip and palate (NSCLP), nonsyndromic hearing impairment, otitis media, preeclampsia, primary open angle glaucoma, inflammatory bowel disease and reumathoid arthritis. The study groups ranged from one to eighty families of varying size. They all used a combination of genetic methods like WGS, WES, sanger sequncing, GWAS and linkage-analysis. Some also used identity by descent analysis (IBD). Only three studies did WGS and three studies made use of family trios. One study searched for rare CNVs instead of SNPs [41]. 23 of the studies found one or a few variants (<5) specific for a family and the rest found several variants without finding one causal variant. Study details on disease, sample, techniques and findings are listed in Additional file 1: Table S1.

Four studies performed a follow-up analysis in unrelated cases and controls and presented an OR. Wetzel-smith et al. detected a missense variant (rs137875858 in *UNC5C)* in a large family with 8 LOAD cases using WGS, WES, linkage-analysis and assay genotyping. The same variant was found in four other pedigrees. Further genotyping of LOAD cases (8,050) and controls (98,194) resulted in an OR = 2.15 (95% CI = [1.21; 3.84], *P* = 0.0095) [42]. By WES and genotyping Cruchaga et al. detected a rare variant (rs145999145 in *PLD3)* segregating in 2 pedigrees with multiple LOAD cases. When testing independent cohorts of sporadic cases (4,998) and controls (6,356) they found an OR = 2.10 (95% CI = [1.47; 2,99] *P*=2.39×10^−10^-). For familial LOAD cases (1,106) vs. unrelated controls (6,356) an OR of 3.39 (95% CI = [2.14; 5.39] *P* = 1.18×10^−6^) was calculated [36]. Kohli et al. made use of WES, linkage analysis, genotyping and Sanger sequencing to study one pedigree counting 15 individuals. They found a rare variant (rs377155188 in *TTC3*) which segregated perfectly with LOAD in the pedigree. They calculated an OR for LOAD of 3.35 for sporadic cases (6,669) vs. controls (5,585) [43]. The result did not reach statistical significance (CI not specified). Goes et al. exome sequenced 36 individuals from 8 families and calculated ORs for variants in three genes with association to bipolar disorder in a case–control follow-up study (3,541 cases, 4,774 controls). It resulted in ORs of 2.73 (*P* = 0.016), 6.7 (*P* = 0.0039) and 2.78 (*P* = 0.045) for variants in *MLK4, APPL*2 and *HSP90AA1* respectively [44].

Some studies found complete segregation between a variant and the studied disease and a few of them will be highlighted in the following. Cruceanu et al. exome-sequenced DNA from caucasian individuals affected with a highly heritable subtype of bipolar disorder in multigenerational families with three to seven affected individuals. Focus was on variants with MAF<1% in the general population. A missense variant was detected on chromosome 11 (position116652892) that leads to substitution of an aminoacid in the encoded protein. The variant segregated with affected family members in a family (five individuals) and was not present in unaffected family members selected as controls (six individuals). Whether this variant contributes to bipolar disorder is not known because of lack of knowledge about the involved gene [37].

Nyegaard et al. studied a five generational family containing 17 individuals with nonsyndromic hearing impairment (two of them dead). Seven already known hearing loss genes were not involved. 11 individuals were selected for SNP genotyping. 11.034 detected SNPs were included in a parametric linkage analysis which resulted in a significant linkage peak at chromosome six. To narrow down the size of the detected locus, 26 family members were genotyped for seven microsatellite markers. Then DNA from an affected individual was selected to undergo next generation sequencing (NGS) at the locus site by a costume designed sequence array. This identified 28,300 variants. One variant found in a coding region and predicted to have a functional effect was identified after excluding common variants. The mutation c.574C>T in *CD164* was found in all affected individuals including a young girl with signs of a beginning hearing loss. The variant was absent in 12 unaffected family members, in one with unknown phenotype and in 1200 unrelated controls [45].

## Suggested application of the family approach to migraine

The studies reviewed here largely followed the steps we have described, but with many variations. In our description of a family based study design, we suggest to use family trios for WGS. Only three studies [46–48], one of them still ongoing, did this. Also, only three studies [42, 46, 49] made use of WGS, which probably reflects that the cost of WGS has only been manageable very recently. It is highly likely that the non-coding regulatory areas play an important role in common migraine [70]. Georgi et al. chose to study a pedigree in an isolated population which has also been suggested as a possible approach to find rare variants [16, 46, 71, 72]. 23 studies [36–38, 41–45, 48–62] reported the finding of less than five family specific variants (not all significant). Some studies could not find one specific causal variant probably because disease susceptibility is caused by more than one rare variant specific to a family (oligogenic inheritance). Detection of variants only makes sense if it is followed by further studies to clarify the causality of the variant. An et al. found several variants associated with autism spectrum disorder. They found enrichment of rare causal variants in key neurobiological processes, and overrepresentation of the rare causal variants in functions involving neuronal development, signal transduction and synapse development [48].

In the future, combinations of gene variants might be analyzed by “omics” approach, where bioinformatics integrate genomics, epigenomics, transcriptomics, proteomics and metabolomics [73, 74]. We excluded Ratnapriya et al. [75] from the reviewing part of this study because they studied a rare subtype of macular degeneration. They found a rare variant in a family with early onset macular degeneration in FBN2, but also a common variant in the same gene with a modest association to AMD cases. It is an excellent example of how both rare and common variants in a single gene can contribute to complex forms of a disease phenotype and the understanding of its pathophysiology.

Few studies focused on CNV’s, and only Van Den Bossche et al. [41], studying schizophrenia, succeeded in finding a CNV associated with disease. Rare inherited CNVs were more frequent in familial schizophrenia than in an unaffected control cohort [76]. This supports CNVs as an area of interest when searching for rare disease variants in migraine. In the future, NGS methods will be able to capture CNVs [77].

As mentioned, ORs for SNPs associated with migraine found through a GWAS ranged between 0.85 and 1.24. In the studies using a family approach reviewed here the ORs in follow-up case–control studies ranged between 2.10 and 6.7, the last one for the association between a variant in *APPL2* and bipolar disorder. Like migraine, bipolar disorder and schizophrenia are common and complex neurological disorders with a clearly heritable factor and a diagnosis based on history in the absence of a biomarker. Success in these disorders raises hope to find specific rare variants with high relative risk in migraine families. Jiang et al. reported the preliminary finding of six novel rare non-synonymous mutations in a Chinese family with clustering of migraine without aura using WES. They included four cases (a father and three children) and four unrelated controls. However, the study had several limitations [78]. As far as we know, Jiang et al. is the only study using a family approach and NGS in migraine that has been published. F. Michael Cutrer, Mayo Clinic, Rochester has carried out WES in two large migraine families, according to a published grant description [79]. Five candidate genes were found to segregate with MA in one family. In the other family, including individuals with varying phenotypes, a single variant was detected. Whether the variants are rare was not stated. These results have unfortunately not been published. A similar study is ongoing on at the Danish Headache Center. This project aims to find rare genetic variants conferring a high risk of migraine using a family approach exactly as described previously. Extended Danish families with MA or MO are included. The study is still collecting data and biological material for sequencing and genotyping. A family approach in migraine will encounter obstacles. Correct phenotyping cannot avoid that unaffected controls may develop migraine later. The probability that some affected family members do not carry a family specific variant is high due to the high prevalence of migraine in the general population. Also, unaffected carriers are a possibility due to low level of penetrance. This will complicate the analysis. Many families contain a mix of individuals with MO, MA or so called MAMO (co-occurring MA and MO). It is not known whether the two phenotypes are part of a spectrum of the same disease or different diseases. Taking these problems into account, we still believe that a family approach is the best way to find variants with a high relative risk. Such variants can be the key to understand the pathophysiological mechanisms of migraine, and much more so than the common variants discovered by GWAS. It is obvious that migraine is highly genetically heterogeneous. Pathophysiology as well as response to prophylactic drugs vary considerably [80, 81]. On the other hand, 80% of patients respond to injection of Sumatriptan suggesting the existence of a final common pathway [82, 83]. If the etiology of just a few sub-phenotypes can be identified with certainty, it seems possible to identify one or more migraine pathwaysthat may be relevant for many patients, even if the genetic cause that lead to the discovery is rare. New targets for better and more specific treatments may then be discovered.

## Review and conclusions

It has proven possible to find rare high risk variants for common complex diseases through a family based approach. One study using a family approach and NGS to find rare variants in migraine has already been published but it has strong limitations. More studies are under way.

Future family approach studies could be advanced by choosing isolated populations or individuals with severe phenotypes as study groups and include analysis of mitochondrial DNA and “omics”.

## Additional files

Additional file 1: Table S1. Summary of family studies. Statistical measures are stated where possible. (DOCX 64 kb)

## Abbreviations

AMD: Age-related macular degeneration
ASD: Autism spectrum disorder
CNV: Copy number variant
GWAS: Genome wide associations study
IBD: Identity by descent
LOAD: Late onset Alzheimer’s disease
LOD: Logarithm of the odds
MA: Migraine with aura
MAF: Minor allele frequency
MAMO: Migraine with and without aura
MO: Migraine without aura
NGS: Next generation sequencing
NSCLP: Non syndromic cleft lip palate
SCZ: Schizophrenia
SNP: Single nucleotide polymorphism
WES: Whole exome sequencing
WGS: Whole genome sequencing
