# Supplementary material for: Family studies to find rare high risk variants in migraine
Source: J Headache Pain. 2017 Mar 2;18(1):32. doi: 10.1186/s10194-017-0729-y (PMC5334193; doi:10.1186/s10194-017-0729-y)
Supplement: Additional file 1: Table S1. — Summary of family studies. Statistical measures are stated where possible. (DOCX 64 kb) [file 10194_2017_729_MOESM1_ESM.docx]

Supplementary Material

Supplementary table 1. Summary of family studies. Statistical measures are stated where possible.

| Study | Disease/trait | Sample | Techniques | Results |
| --- | --- | --- | --- | --- |
| Bowden et al. [56] | Adiponectin level | 80 families | Linkage-analysis, WES, Sanger sequencing. | The G45R variant in the *ADIPOQ* gene was identified in 2 families with lower mean plasma levels of adiponectin. The allele frequency was 18% in the 2 families versus 1.1% in the cohort. A total of 27 individuals from 7 families, showed to have the variant. |
| Yu et al. [38] | Age-related macular degeneration (AMD) | 9 families (42 DNA samples) | WES, genotyping by bead-array, Sanger sequencing | One family with the R53C variant: LOD= 5.07 (*P*= 6,7$\times$ 10^-7^)  One family with D90G variant:  LOD score= 1.22 (*P* = 0,009) |
| Weeke et al.[92] | Atrial fibrillation | 6 families | WES, Sanger sequencing | In 5 families, 7-15 very rare variants in 28 different genes were detected. One specific disease causing variant was not found in any of the families. |
| An et al.[48] | Autism spectrum disorder (ASD) | 40 trio or sibling families (48 affected, 80 controls) | WES | Were looking for both de novo and inherited variants and several were identified. Most promising was that in 4/48 cases, inherited variants in the *L1CAM* interaction pathway were found. |
| Cukier et al.[93] | Autism spectrum disorder (ASD) | 40 families (164 individuals in total, 105 affected, 59 unaffected) | WES, SNP genotyping, Sanger sequencing, IBD analysis | 32 rare SNPs, not previously associated with ASD, segregated in ≥ 2 families. |
| Shi et al.[91] | Autism spectrum disorder (ASD) | 1 family (10 individuals, 2 affected) | WES, SNP genotyping, Sanger sequencing, IBD analysis | 7 candidate genes with rare variants shared by the 2 affected individuals were detected. Whether they are disease causing was not confirmed. |
| **Cruceanu et al.** [37] | Bipolar disorder | 25 families analyzed | WES, Sanger-sequencing | A rare missense variant (position 116652892) on chromosome 11 found to segregate in a family. The variant was not present in unaffected family members. |
| Egawa et al.[59] | Bipolar disorder | 1 family (2 affected twins, the mother and 2 brothers were unaffected, father was undiagnosed but had symptoms) | WES, Sanger sequencing, genotyping assay | Two variants *WDR90* V1125fs and *EFCAB5* L1210fs, identified to be transmitted from the father to a monozygotic twin pair. Variants were absent in unaffected brothers. They were not redetected in a follow-up study in unrelated individuals. |
| Georgi et al. [46] | Bipolar disorder | 388 individuals from a large (Old Order Amish) pedigree with many nuclear families. WGS of 50 individuals (23 affected) | GWAS, WGS, Linkage-analysis | A number of candidate-genes found to be shared by 3 linked families. A single causal gene or variant was not found. |
| Goes et al.[44] | Bipolar disorder | 8 families (6.9 affected on average, 55 individuals in total) | WES | 84 segregating rare variants were found in 82 genes.  Follow up in 3 case-control samples (a total of 3,541 cases and 4,774 controls) resulted in detection of 3 variants with significant association and OR >1. Genes and the OR for the 3 variants are listed below.  *MLK4* with OR = 2.73 (*P* = 0.016)  *APPL*2 with OR = 6.7 (*P* = 0.039) *HSP90AA1* with OR = 2.78 (*P* = 0.045) |
| Matoba et al. (ongoing) [47] | Bipolar disorder | 50 trio families | WES | Preliminary analysis showed approximately 43 variants on average to be transmitted to the proband (the child) in 7 families. |
| Strauss et al. [53] | Bipolar disorder | 4 pedigrees with a total of 26 family members (14 affected) | WES, genotyping by SNP array | The rs78247304 variant in the *KCNH7* gene was found by WES and filtering of DNA from 7 individuals with bipolar disorder from 4 different families. The finding was not genome wide significant. |
| Tanaka et al.[63] | Diabetes (Type II) | 1 family (16 living individuals, 13 affected) | Linkage-analysis, WES, SNP genotyping | 7 rare variants were found to segregate with affection status in the pedigree. One of them, N1072K in *EEA1*, was found to be significantly more frequent in 67 lean diabetes subjects than in 105 normoglycemic controls (*P* = 0.022, Fishers exact test). p.N1072K was further detected in 3/64 unrelated familial diabetes cases (MAF = 2.3%). |
| Prudente et al. [55] | Diabetes (Type II) | 60 families | WES, Sanger sequencing | A nonsense variant in *APPL1* found to segregate in an Italian family (counting 28 individuals, 10 of them affected,10 unaffected carriers). A missense variant in *APPL1* found to segregate in an American family (counting 12 individuals, 5 of them affected, 1 unaffected carrier). |
| Okou et al.[64] | Inflammatory bowel disease | 1 family (5 individuals, 4 affected) | SNP-genotyping, WES, Sanger sequencing | A missense variant, c.694A>C in *FOXP3* on chromosome X identified to be inherited from an affected mother to her 3 affected sons. |
| Farlow et al.[94] | Intracranial aneurysm | 7 families | Linkage-analysis, WES, | A total of 68 rare exonic variants in 68 genes were identified in the 7 families. 8 of them segregated with all aneurysmal phenotypes and were not carried by unaffected individuals. The variants were found in following genes: KLF11, ABCC3, TANC2, ALMS1, ARHGEF1, SMEK2, HTRA2, and NDST1. |
| Cruchaga et al. [36] | Late onset alzheimers disease (LOAD) | 14 families (WES on 29 affected and 11 unaffected individuals) | WES, genotyping | A rare variant in *PLD3* gene (rs145999145) segregating in 2 pedigrees.  The variant was associated with LOAD risk.  OR = 2.10 in 4,998 sporadic cases vs. 6,356 controls (95% CI = [1.47; 2,99] *P* = 2.39$\times$10^-10^-)  OR = 3.39 (95% CI = [2.14; 5.39] *P* = 1.18 $\times$10^-6^) in 1,106 familial cases vs. 6,356 controls not related to the cases. |
| Kohli et al.[43] | Late onset alzheimers disease (LOAD) | 1 family with 15 affected (DNA samples from 11 affected and 5 unaffected) | WES, linkage analysis, genotyping by bead array, Sanger sequencing | A rare variant in the *TTC3* gene (rs377155188) segregated perfectly with LOAD in the pedigree. 1 unaffected carrier of 5 tested. Further study in a case-control dataset (6,669 cases, 5,585 controls) resulted in OR 3.35 (did not reach statistical significance. CI not specified). |
| Saad et al.[95] | Late onset alzheimers disease (LOAD; age at onset) | 77 individuals from families with multiple affected (inclusion of up to 4 affected per. family) | GWAS, linkage-analysis, WES, IBD analysis | Several candidate genes detected. Found one SNP (rs2291516, MAF = 0.08, P value = 4.12 × 10^−7^) with Bonferroni-corrected significance (0.05/39 993 = 1.25 × 10^−6^) in the gene RGL3. Further 14 SNPs with higher *P*-values were found in 11 different genes. |
| Wetzel-Smith et al. [42] | Late onset alzheimers disease (LOAD) | 1 pedigree with 8 affected (further analysis on 863 probands from other families and linkage-analysis in 4 of these families) | Parametric linkage analysis, WGS, WES, genotyping assays | A rare variant  (rs137875858) in *UNC5C* gene segregating in a pedigree. The variant was present in all cases with available DNA (6) and 2 unaffected. Further analysis on 863 other probands resulted in 4 other cases positive for the variant of which one showed to segregate with cases in the family. OR = 2.15 in unrelated 8,050 cases vs. 98,194 controls (95% CI = [1.21;3.84], *P* = 0.0095) |
| Aylward et al.[60] | Nonsyndromic cleft lip and palate (NSCLP) | 25 families | WES, Sanger sequencing | Identification of variants in 4 genes present in ≥3 families. Variants in 2 of the genes, *ACSS2* and *PHYH,* segregated with NSCLP as a dominant variant with incomplete penetrance. |
| Nyegaard et al. [45] | Nonsyndromic hearing impairment | One family (29 living individuals, 15 affected) | GWAS, linkage- analysis, NGS only at the locus side by using a custom designed sequencing array. | The mutation c.574C>T in *CD164* was found in all affected individuals. LOD-score of the locus 5.1. |
| Santos-cortez et al. [57] | Otitis media | A pedigree consisting 134 individuals (from an intermarried, indigenous Filipino community). (DNA from 51) | WES, Sanger sequencing. | A duplication in the *A2ML1* gene (c.2478_2485dupGGCTAAAT (p.Ser829Trpfs*9) possibly segregated with the disease in the pedigree. LOD-score 7.5. |
| Deng et al. [58] | Parkinsons disease | 1 family with 81 members, 15 affected (DNA samples from 65) | Likage analysis, WES, Sanger sequencing | A rare missense variant in *TMEM230* were present in the DNA from the 13 affected individuals tested. It is unclear whether they found unaffected carriers. |
| Johnson et al.[61] | Preeclampsia | 2 families (18 individuals in total, 7 affected, 11 controls) | WES | A rare SNP (rs111033530) residing within the *GPR98* gene was detected in 1 family. |
| Liu et al.[96] | Primary open angle glaucoma | 1 family (16 affected) | WES | Several mutations in CD2, PKHD1, PAH and FUT7 were identified. |
| Okada et al.[62] | Rheumatoid arthritis (RA) | 1 pedigree (49 individuals, 8 affected, 10 deceased) | GWAS, IBD mapping, WES | A rare non-synonymous mutation in PLB1 (p.G755R) were found to co-segregate with RA, showing a dominant inheritance with incomplete penetrance. It was present in all 5 tested cases and in 7 of 16 unaffected (P  =  0.009). |
| Homann et al. [97] | Schizophrenia (SCZ) | 9 families (90 DNA samples) | WES | 25 rare SNPs and a deletion detected, each unique, to one of the 9 families, and present in all the sequenced affected members in that family. Some of them also present in controls.  Several of the genes involved have previously been found to have association with neurodevelopment and schizophrenia. |
| Thygesen et al. [49] | Formal thought disorder/schizophrenia (SCZ) | 6 pedigrees = a total of 618 individuals. (DNA samples from 256) | Genotyping of SNPs and microsattelites by bead-array, linkage-analysis, WGS, Sanger sequencing | A haplotype on chromosome 6 found to segregate with thought disturbances in 31 individuals in a pedigree with max-LOD score of 4. WGS on three of these individuals detected a single nucleotide deletion (chr6: 1643777205 AG>A). |
| Timms et al. [54] | Schizophrenia (SCZ) | 5 families (DNA available for 24 affected and 17 unaffected) | WES, Sanger sequencing, GWAS for linkage analysis | 4 different variants found to segregate in 4 families in 3 genes associated with the NMDA-receptor. |
| Van Den Bossche et al. [41] | Schizophrenia (SCZ) | 8 families | Bead chip CNV analysis, multiplex amplicon quantification | One CNV found in 6 family members in a family, of which 4 had schizophrenia related disorders. 2 CNVs found in another family. One of them in the mother who had symptoms but no disorder, and 2 in affected children. The other was found in the healthy father and all three affected children. |
| Zhou et al. [65] | Schizophrenia (SCZ) | 1 family (12 individuals, 6 affected) | Linkage-analysis, WES, Sanger sequencing | A rare missense variation c.9575 C > G (p.Thr3192Ser) in *RELN*, which is known as a risk gene for SCZ, was indentified in affected individuals and absent in unaffected. It was not detected in the 500 unaffected control individuals. |
